# Analysis of the geometrical influence of ring-opening samples on arterial circumferential residual stress reconstruction

**DOI:** 10.3389/fbioe.2023.1233939

**Published:** 2023-08-22

**Authors:** Matías Inostroza, Andrés Utrera, Claudio M. García-Herrera, Eugenio Rivera, Diego J. Celentano, Emilio A. Herrera

**Affiliations:** ^1^ Departamento de Ingeniería Mecánica, Universidad de Santiago de Chile, Santiago, Chile; ^2^ Departamento de Ingeniería Mecánica y Metalúrgica, Pontificia Universidad Católica de Chile, Santiago, Chile; ^3^ Pathophysiology Program, Faculty of Medicine, Institute of Biomedical Sciences (ICBM), Universidad de Chile, Santiago, Chile; ^4^ International Center for Andean Studies (INCAS), Universidad de Chile, Santiago, Chile

**Keywords:** residual stress, biomechanical characterization, soft tissues biomechanics, numerical simulation, ring opening

## Abstract

This work consists of analyzing the impact of geometrical features (thickness and curvature) on the estimation of circumferential residual stresses in arteries. For this purpose, a specific sample of lamb abdominal artery is chosen for analysis and, through computational tools based on Python libraries, the stress-free geometry is captured after the ring opening test. Numerical simulations are then used to reconstruct the sample in order to estimate the circumferential residual stresses. Then, four stress-free geometry models are analyzed: an ideal geometry, i.e., constant curvature and thickness; a constant curvature and variable thickness geometry; a variable curvature and constant thickness geometry; and a variable curvature and thickness geometry. The numerical results show that models perform well from a geometric point of view, where the most different feature was the closed outer perimeter that differs about 14% from the closed real sample. As far as residual stress is concerned, differences up to 198% were found in more realistic models taking a constant curvature and thickness model as reference. Thus, the analysis of a realistic geometry with highly variable curvature and thickness can introduce, compared to an idealized geometry, significant differences in the estimation of residual stresses. This could indicate that the characterization of arterial residual stresses is not sufficient when considering only the opening angle and, therefore, it is also necessary to incorporate more geometrical variables.

## 1 Introduction

It has been long addressed that blood vessels are not stress-free in a physiological state (Vaishnav et al., 1983; Chuong and Fung, 1986; Fung and Liu, 1989), stating that the stress distribution along the arterial wall is relevant in both normal and pathological conditions (Okamoto et al., 2002; Guo et al., 2005), and plays an important role on the pressure-strain distribution along the vessel wall (Chandran et al., 2001). Moreover, some of these studies that deal with the biomechanics of cardiovascular diseases or experimental treatments measure the residual stress distribution to observe a possible alteration in the homeostatic condition of the tissues or to assess the mechanism of disease progression (Okamoto et al., 2002).

The ring-opening test is one of the most commonly used methods to characterize the residual stresses in arteries (Fung and Liu, 1989). This experimental test consists of making a radial cut on a vessel, measuring the angle formed by the artery ends and the midpoint of the wall perimeter. Several authors have used this simplified method to describe the residual circumferential stress of many arterial tissues (Haghighi et al., 2004; Haghighipour et al., 2010; Sokolis, 2015; García-Herrera et al., 2016; Sokolis, 2019; Navarrete et al., 2020; Zhuan and Luo, 2020). Although the ring-opening test is very popular and straightforward to carry out, it has been reported that the residual stress problem is very complex and non-homogeneous (Holzapfel et al., 2007; Badel et al., 2012), meaning that the assumption of a unique parameter of deformation cannot describe the entire aspects involved in the test. Nevertheless, the ring-opening angle method is still easy to measure the approximate residual strains on a tubular sample.

However, non-uniform ring opening geometries have been reported. In this context, Han and Fung (1991) have reported differences in the opening angles in the aortic arch the depending on the polar angle of the radial cut. This suggests that different levels of residual strain can be found within a single sample in curved zones. Besides this, the study of Delfino et al. (1997) suggests that the residual strain can also highly vary in the vicinity of bifurcations, such as carotid bifurcation. Also, several experimental reports of opened arterial tissues do not accurately represent the uniform opening shape assumed by the ring-opening test. According to Okamoto et al. (2002), 21 out of 55 ring opened samples from his study of ascending aorta dilatation were excluded due to the non-uniformity of the samples. Holzapfel et al. (2007) made a residual strain analysis from human aortas rings (entire samples and layer separated) and concluded that residual deformations are three-dimensional phenomena and cannot be characterized by just the opening angle, besides geometrical parameters such as the curvature or thickness, as they can vary along the ring perimeter. In thoracic aorta aneurysms, Sokolis (2015) has described the difficulties of analyzing residual strains due to the non-uniform opening shapes obtained in his research, establishing the existence of a spatial dependency of the strain fields caused by this pathology. (Fung and Liu, 1989) radially cut a sample into four segments, aiming to show that one cut releases all the residual stress. Although the hypothesis was successfully verified, the geometry changes exhibited within each segment, such as curvature differences, were not analyzed. Sigaeva et al. (2019) has proposed a method that consists of assessing the non-uniform geometries with a multi-sector approximation, in which the entire ring shape is mathematically discretized into different arc sectors, calculating the stretches and stresses in each one of them, with both theoretical and numerical approaches. Ohayon et al. (2007) have investigated the effects of residual strains and stresses in tissues with vulnerable plaque, using patient-specific geometries produced by the opened shape of the diseased tissues to evaluate the stress field using a finite element analysis. Finally, Heming et al. (2021) have studied the characterization of the residual stresses of a porcine aorta from its proximal to the distal end. The author sliced a series of rings along the artery, and used the opened geometry as an initial condition in a finite element analysis, in which the ends of the geometry are subsequently closed with a displacement-based approach (assuming a stress-free opened configuration), thus allowing the reconstruction of the stress fields in the closed geometry. As all of these authors suggested, that kind of analysis requires more realistic considerations to be included in a non-patient-specific study.

The main aim of this study is to analyze the effect of the non-uniform opened geometry of arterial samples, considering both thickness and curvature variations along the circumference of the cut ring sample to obtain the residual stress and strain fields of the closed ring shape, using both experimental and numerical approaches, and considering only passive and time-independent response of the tissue. The overall experimental procedure consists of performing a typical opening process, taking a photography of the opened geometry. Then, the approximate 2D geometry of the transverse plane is recreated, fitting a spline function in both the inner and outer contours of the sample. This geometry is considered in a finite element simulation that is carried out afterward. To this end, the resulting sample is meshed to perform a displacement-based closing procedure, aimed at connecting both ends of the sample, to obtain a correlation between the numerical results with the specific geometrical descriptors of the sample. Finally, a pre-stretched pressurization simulation is computed in order to evaluate the effect of local geometry variations in a pressurized vessel.

## 2 Materials and methods

This study was approved by the Institutional Animal Care and Use Committee (IACUC) of the University of Chile (Protocol CBA 0761 FMUCH). All the animal procedures were carried out following the Guide for the Care and Use of Laboratory Animals published by the US National Institutes of Health (NIH Publication No. 85-23, revised 1996) and the ARRIVE Guidelines (Kilkenny et al., 2010).

This work takes as reference the study by Rivera et al. (2020), which consists of the passive mechanical characterization of the aortic artery of lambs born and raised under conditions of chronic hypoxia (3,600 m above sea level) with and without melatonin application. As the subject of analysis, emphasis is placed on a specific sample of the abdominal zone of a control group lamb (33 days old and 9.1 kg weight at the time of euthanasia with injection of sodium thiopental, 100 mg kg^−1^), whose stress-free configuration (after the ring-opening test) presented a highly variable curvature, so that the geometric assumptions of the most widely used methodologies (Chuong and Fung, 1986; Sigaeva et al., 2019) lose representativeness of the physical phenomenon.

### 2.1 Tensile test

This test is aimed at obtaining the material behavior under a uniform strain regime in order to characterize the constitutive model described in Section 2.2. During the experiment, the test is performed under physiological conditions, maintaining the tissue samples submerged in calcium-free saline solution at 39°*C* [physiological range for lambs (Rivera et al., 2020)]. The samples were obtained from arteries (specifically from the abdominal aorta) by cutting rectangular strips in the longitudinal and circumferential directions of the vessel. The resulting strips were stretched in an Instron 3342 universal testing machine equipped with a 10 N load cell, with force and displacement measurement precision of ±0.01N and ± 1 μm, respectively. The stretch was imposed at a constant speed of 1.5 mm/min, and no preconditioning was applied to the samples.

In each test, the Cauchy stress is calculated as $\sigma =\frac{F}{A}$ , where *F* is the force measured by the load cell, and *A* is the instantaneous cross-sectional area of the sample. The stretch during the test is calculated as $\lambda =\frac{l}{l_{0}}$ , where, *l* is the instantaneous length of the sample and *l*_0_ is the initial length. Due to the incompressibility assumption, the instantaneous cross-sectional area can be calculated as $A=\frac{A_{0}}{\lambda }$ , where *A*_0_ is the initial cross-sectional area, measured adequately before the test.

### 2.2 Constitutive modeling

An elastic and rate-independent material model is considered to analyze the behavior of the studied case in the application of the proposed closing methodology to be presented in Section 2.5. To this end, the anisotropic strain energy function introduced by Gasser et al. (2006), denoted as HGO from here onwards, is adopted in the present work to model the behavior of arterial tissues with distributed collagen fiber orientations.

The strain energy function *W*, assumed to describe the isothermal behavior of the material under any loading conditions, can be defined in terms of the right Cauchy deformation tensor **C** = **F** ^*⊤*^ **F**, where **F** is the deformation gradient tensor. It is possible then to obtain the Cauchy stress tensor as $\sigma =2J^{-1}F\cdot \frac{\partial W}{\partial C}\cdot F^{⊤}$ , where *J* represents the volume change, calculated as the determinant of the right Cauchy green tensor. The strain energy function is defined by the following expression:

$$W=\frac{\mu }{2}\left({\bar{I}}_{1}-3\right)+\frac{k_{1}}{2k_{2}}\underset{\alpha =4,6}{\sum }\left[\exp\left(k_{2}{\left({\bar{I}}_{1}\kappa +\left(1-3\kappa \right){\bar{I}}_{\alpha }-1\right)}^{2}\right)-1\right]+\frac{k}{2}{\left(\ln J\right)}^{2}$$ (1)

where *μ*, *k*_1_, *k*_2_ and *κ* are material parameters, such that *κ* is associated with the fiber dispersion, and whose value can vary between 0 and 1/3. ${\bar{I}}_{1}$ is the first invariant of $\bar{C}=J^{-2/3}C$ , while ${\bar{I}}_{4}$ and ${\bar{I}}_{6}$ are defined as ${\bar{I}}_{4}=a\cdot \bar{C}\cdot a$ and ${\bar{I}}_{6}=a^{\prime }\cdot \bar{C}\cdot a^{\prime }$ , where *a* and *a*′ are unit vectors, described in the reference configuration, symmetrically disposed at angles ± *φ* with respect to the axial axis of the vessel, accounting for the orientations of two collagen fiber families. Finally, *k* is associated with the bulk modulus of the material, enforcing quasi-incompressibility during the finite element analysis.

This model is implemented in an in-house finite element software extensively validated in many biomechanics pieces of research, where the implementation considers that the contribution of the collagen fibers is present only in tension (Gasser et al., 2006). Moreover, an improved strain-displacement matrix for large strains, designated B-bar element formulation, is implemented to avoid numerical locking due to material incompressibility (Celentano, 2001).

Finally, the material parameters are obtained through a least-squares optimization of the analytical stress expression of the HGO model against the experimental strain-stretch curves obtained via the longitudinal and circumferential uniaxial tensile tests.

### 2.3 Ring-opening test

**FIGURE 1:**
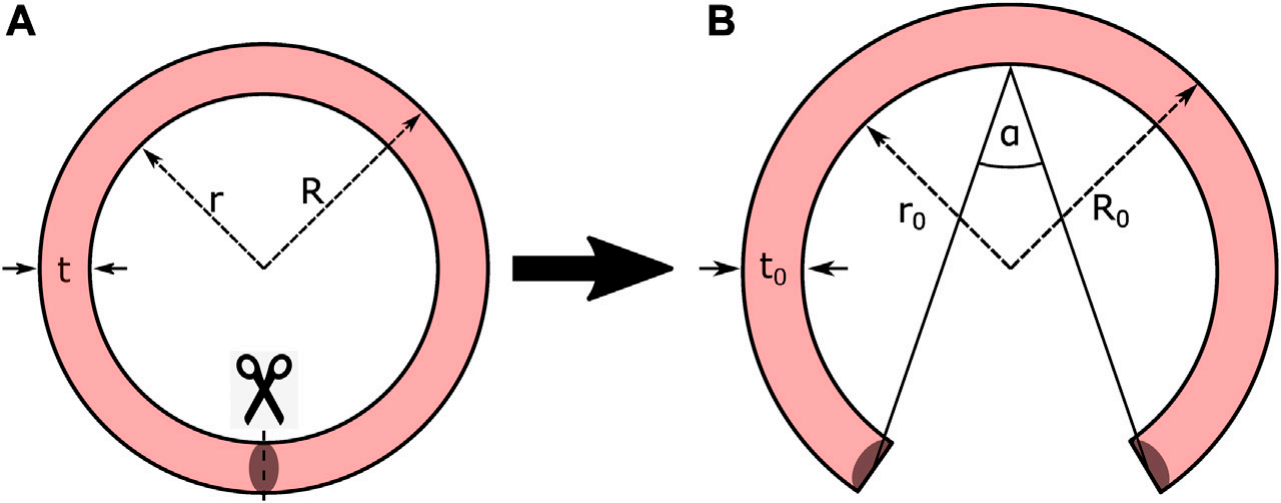
Idealized ring opening test protocol. **(A)** Closed geometry; and **(B)** Stress-free configuration.

In this test, the artery sample is extracted from the abdominal region of the aorta by making a cut in its transverse plane using a scalped blade, thus getting an approximate ring specimen length of 1 mm. After the cut, the specimen is maintained in Krebs for 15 min at a temperature of 39°C [physiological range for lambs (Rivera et al., 2020)]. Figure 1 schematically describes the ring-opening test protocol. The ring specimen is positioned in a Petri dish filled with Krebs buffer under a magnifying glass (Motic SMZ-161) and photographed with a digital camera (Motic Moticam 2.0 MP). Then, a radial cut is made to the sample in the opposed position to the column of the animal, identified with a black reference point in the tissue, which is marked before being extracted from the animal’s body. Once the tissue releases its elastic energy, the open shape of the sample is obtained, and it is kept submerged in Krebs for 15 more minutes to dispense with viscoelastic effects and fully release the residual stress (Fung and Liu, 1989; Han and Fung, 1991). After that, a new photograph of the sample is taken, ensuring that the transverse plane of the ring be as parallel as possible to the dish surface and the camera lens.

**FIGURE 2:**
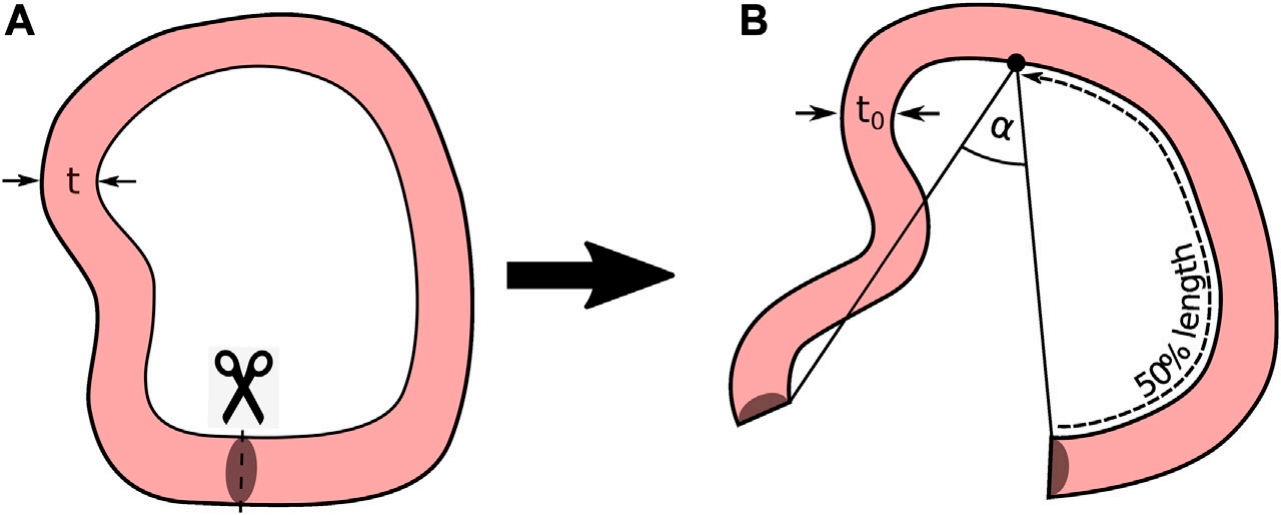
Variable Geometry Ring Opening Scheme. **(A)** Closed geometry; and **(B)** Stress-free configuration.

The typical opening shape represented in Figure 1 assumes that after the cut, the mean perimeter of the closed shape *P* ^*m*^ = 2*πr* ^*m*^ (where *r* ^*m*^ = (*r* + *R*)/2 is the closed mean radius) is equal to the mean perimeter of the opened shape $P_{0}^{m}=2\pi r_{0}^{m}-r_{0}^{m}\alpha$ (where $r_{0}^{m}=\left(r_{0}+R_{0}\right)/2$ is the opened mean radius), such that, this situation allows to easily reconstruct either the strain or stress fields with analytical or numerical approaches. However, using this assumption in geometries that do not have a constant curvature, as those shown in Figure 2, could result in a significant bias from the actual mechanical problem.

The stress distributions in the sample considering the opening angle assumption will be compared through finite element simulations with four different geometries, as described in Section 2.5.

### 2.4 Geometry extraction and meshing

Four types of geometries are analyzed in this study. The first one consists of idealizing the real sample geometry into the classical ring-opening shape (see Figure 1), transforming the measured opening angle and perimeter of the sample in a constant curvature and thickness geometry (Case A). The second geometry is based on the measured thickness, maintaining a constant curvature (Case B). The third and fourth analyses consist in the use of the measured curvature of the sample via image analysis, with the difference that the second includes constant thickness (Case C) and the third accounts for the obtained thickness from the image analysis (Case D). The utilized framework that allows to extract the geometry of the samples allows measuring the thickness and curvatures that describe this geometry in its entire plain domain. This procedure is based on the Python UnivariateSpline library, allowing to fit a parametric spline *s*(*t*) to a series of points, in order to define the inner and outer parametric curves of the analyzed sample that represents the inner and outer walls of the vessel. The spline function is composed of two separated parametric parts that define both *x* and *y* coordinates of the geometry as follows:

$$s\left(t\right)=\left\{\begin{array}{c} x\left(t\right)\;\\ y\left(t\right)\; \end{array}\right.,\;t\in \left[0,t_{\max}\right]$$ (2)

where the maximum *t* value (*t*_max_) is an arbitrary maximum value chosen for the parametric variable.

**FIGURE 3:**
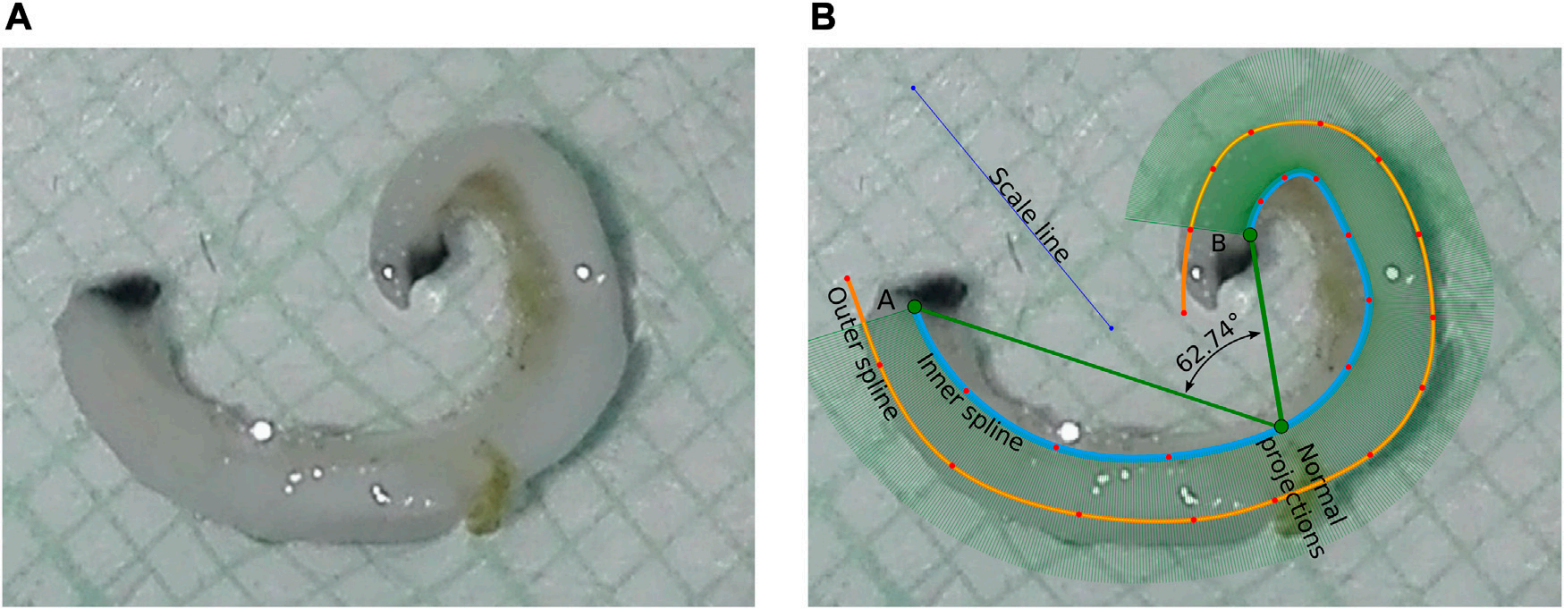
Geometry extraction procedure. **(A)** Opened ring sample **(B)** Contour identification. Size grid equals to 1 mm.

Figure 3 represents the overall geometry extraction procedure. The first step of the method consists on defining the scale line, in order to transform all the measurements to a suitable unit. A series of dots positioned over the inner perimeter of the sample determines the inner spline (light blue spline). The same concept is applied to the outer side of the sample (orange spline). Then, a series of perpendicular projections (dense arrangement of green lines) are made with respect to the inner spline, solving for each one the intersection between the line and the outer spline, aimed at accomplishing two issues: on one side, it is done to define consistent ends in the geometry that are perpendicular to the inner spline at each end and, on the other side, it allows measuring the thickness across the entire geometry, which will be defined as the length of the resulting line from the inner spline to the calculated intersection at the outer spline. It should be noted that the direction of the defined splines will need to remain the same in the finite element analysis in order to compare the numerical results with the measured geometrical variables correctly. The curvature of the geometry is measured at the inner spline, with the following analytical expression:

$$\kappa \left(t\right)=\frac{\left|\dot{x}\ddot{y}-\dot{y}\ddot{x}\right|}{{\left({\dot{x}}^{2}+{\dot{y}}^{2}\right)}^{3/2}}$$ (3)

Finally, from the actual thickness measurements it is possible to obtain a constant-thickness geometry, calculating the average thickness across the geometry, and then perform a perpendicular offset from the inner spline, using the normal vector along with the thickness average to obtain a new outer perimetric curve.

After all the geometries are defined, the dimensions and coordinates from each case of study are used within Gmsh software, in order to mesh an 8-noded hexahedral finite element model.

### 2.5 Numerical simulation procedure

#### 2.5.1 Ring-closing

**FIGURE 4:**
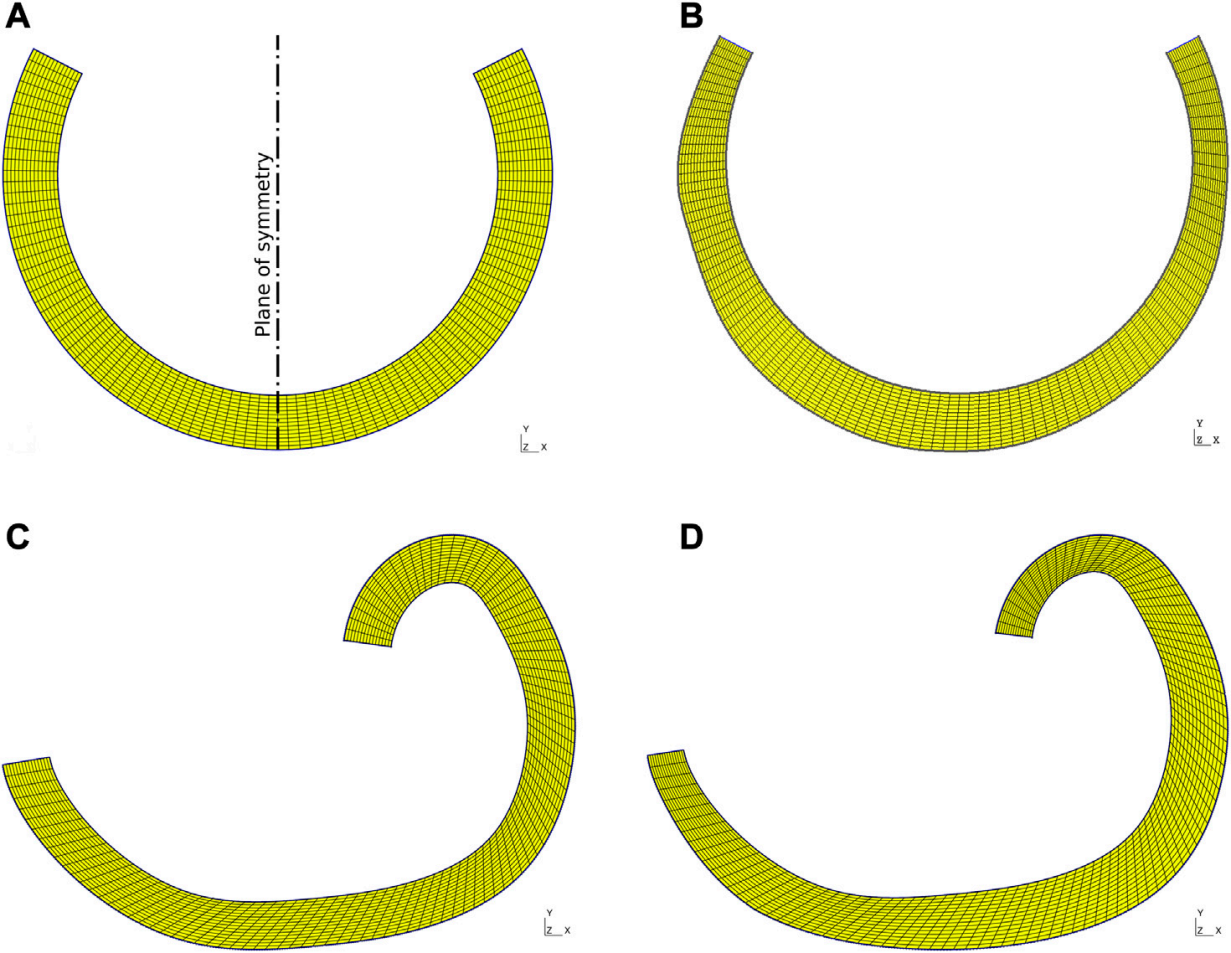
Mesh models **(A)** Case A: Constant curvature and thickness mesh; **(B)** Case B: Variable thickness and constant curvature; **(C)** Case C: Variable curvature and constant thickness mesh; **(D)** Case D: Variable curvature and thickness mesh.

The digitization of the sample allows the modeling of the geometries of the previously discussed analysis cases A, B, C, and D which are represented in Figures 4A–D, respectively.

Regarding the finite element simulations, two computational closed methodologies are identified. Each of them is based on the displacement of one of the faces where the cut was made, while the displacement of the other face is fixed, either totally or partially, depending on the case analyzed.

**FIGURE 5:**
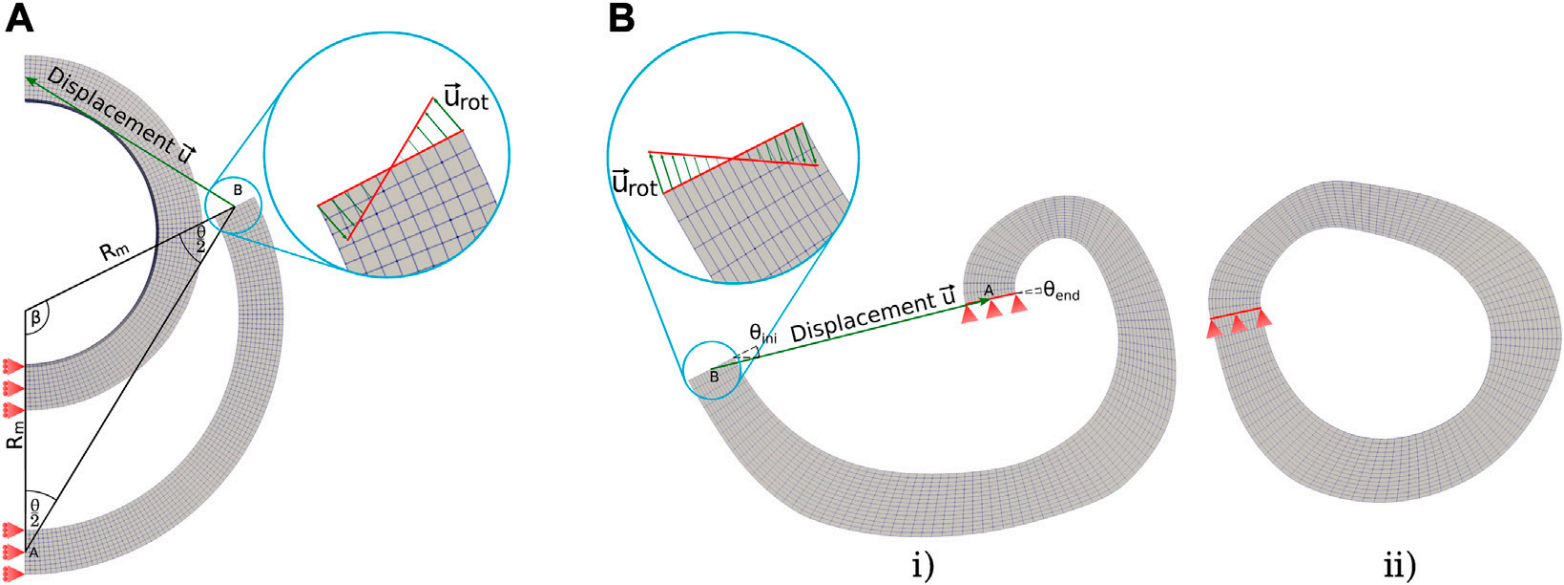
Closing boundary conditions. **(A)** Case A, idealized geometry closing procedure, **(B)** Cases B, C, and D variable geometries’ boundary conditions; i) initial and ii) final positions of the FE model.

• Geometry with constant curvature: Face *A*, corresponding to the plane of symmetry (see Figure 4A), is fixed in the horizontal direction, allowing vertical movement. Meanwhile, the nodes on face B are displaced through the addition of a displacement vector $\vec{u}$ and a rotation vector ${\vec{u}}_{\mathrm{rot}}$ associated with a total rotation angle *u*_*rot*_ = 180 − *β*, where *β* is the angle between the cut face, symmetry face and the circumference center (see Figure 5A). This procedure is an adaptation of the method applied by García-Herrera et al. (2016)
• Geometry with variable curvature and variable thickness: It can be noted that one of the ends of the geometry is fixed in position during the whole simulation, while the displacements of the nodes in the remaining end will be prescribed. A vector sum between a linear displacement vector $\vec{u}$ and the total rotation vector ${\vec{u}}_{\mathrm{rot}}$ related to the rotation value *u*_*rot*_ = (*θ*_*end*_ − *θ*_*ini*_), where *θ*_*ini*_ is the orientation angle of the movable cut face, and *θ*_*end*_ is the orientation angle of the fixed cut face (see Figure 5B).

It is important to highlight that the movement of the cut faces is discretized into a finite number of steps. Each node that composes faces *B* of the constant and variable curvature models (see Figure 5) is displaced through linear movements ${\vec{u}}^{\mathrm{step}}=\frac{\vec{u}}{nsteps}$ and a rotational movement ${\vec{u}}_{\mathrm{rot}}^{\mathrm{step}}=\frac{{\vec{u}}_{\mathrm{rot}}}{nsteps}$ . The latter is applied in such a way that the thickness of the face *B* remains constant in each step.

#### 2.5.2 Pre-stretch and pressurization

Upon completing the closure, the cylindrical structure is pre-stretched longitudinally and internally pressurized through a new simulation that considers a mesh without the cut disconti-nuity, achieving a stretch of 1.6 as reported in the study by Rivera et al. (2020). Since closed geometry is highly variable in curvature variable models, it was necessary to define the following boundary conditions. The model is fixed at the bottom (that means, one of the ends in longitudinal direction), and vertically displaced in upper surface (remaining end in longitudinal direction). Then, a pressure load is applied to the inner mantle at levels of 60, 120, and 150 [mmHg], corresponding to systolic, diastolic and hypertension blood pressure levels, respectively (Rivera et al., 2020). The results were taken from the upper face, ensuring the fixed restriction is far enough to not affect the phenomenon.

## 3 Results

### 3.1 Material characterization

**FIGURE 6:**
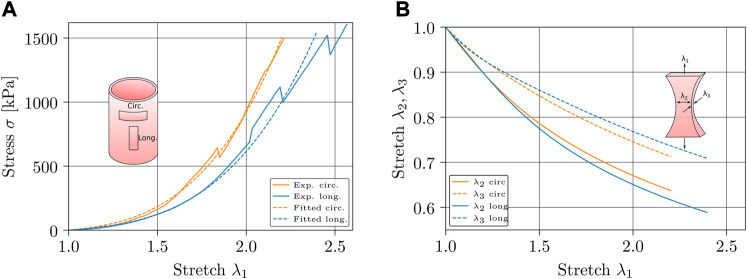
Material characterization. **(A)** Longitudinal (blue) and circumferential (orange) Cauchy stress curves for experimental measurements (continuous lines) and fitted model (dashed lines). **(B)** Numerical transverse stretches of the longitudinal (blue) and circumferential (orange) tensile test, where *λ*_2_ is represented by a continuous line and *λ*_3_ by a dashed one.

To model the mechanical behavior of the material, the parameters of the HGO model are calibrated. For this purpose, a least-square fitting procedure is employed in order to reduce the quadratic errors between the experimental and analytical Cauchy stress of the longitudinal and circumferential directions of the material. Figure 6A presents the longitudinal and circumferential Cauchy stress for the experimental measurements (continuous lines) as well the fitted model (dashed lines). Although there are more sophisticated methods to fit material constants, such as evolutionary strategies (Rivera et al., 2021), the used method provides sufficient reliability to this study, since the physical consistency of the fitted model has been proved, ensuring that the numerical prediction of the second and third stretches (along the sample width and thickness, respectively) of the uniaxial tensile tests responds as expected, see Figure 6B.

It is important to highlight that experimental data considered was taken from the average of six uniaxial tests in both circumferential and longitudinal directions (Rivera et al., 2020). Since samples reach their rupture points at different stretch levels, experimental curves present several stress drops of the average curve.

The fitted parameters are presented in Table 1, where high fitting accuracy can be observed in both longitudinal and circumferential directions. The total normalized root-mean-square deviation (NRMSD) is presented in order to have an absolute error reference for the whole model fit.

**TABLE 1:** Fitted parameters of the HGO model.

*μ*[kPa]	*k*_1_ [kPa]	*k*_2_	*κ*	*φ*°	$r_{\mathrm{long}}^{2}$	$r_{\mathrm{circ}}^{2}$	NRMSD
39.661	145.321	0.02982	0.2599	53.270	0.9642	0.9937	0.0590

### 3.2 Numerical simulations

#### 3.2.1 Ring-closing

As a way of verifying the effectiveness of the computational reconstruction of the three situations considered, geometric aspects are compared between the real geometry of the sample and the three previously specified simulations. The extraction of the geometric parameters of the closed FE model is done through the same technique presented, that is, based on the application of splines on the mesh contour.

**FIGURE 7:**
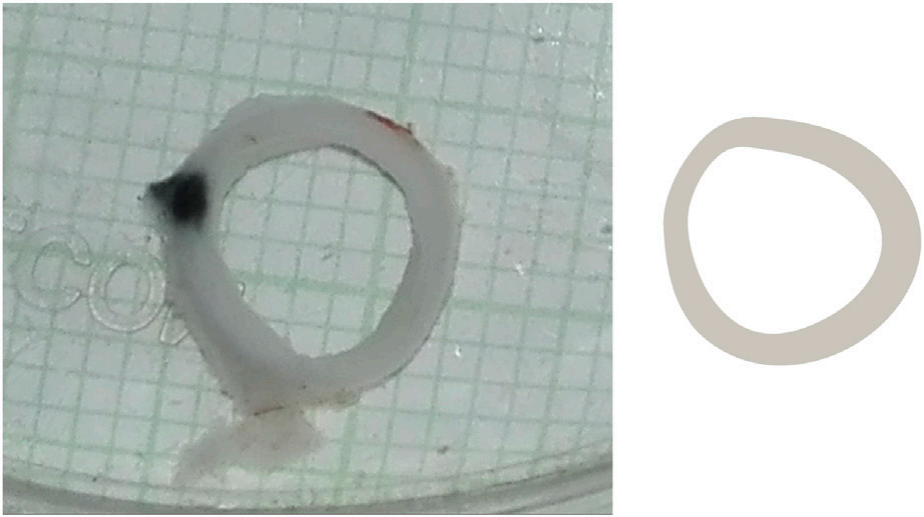
Comparison between real sample (left) and computationally reconstructed geometry (right). Size grid equals to 1 mm.

The results are presented in Table 2, where perimeters are estimated through the length of the internal and external Splines. The mean curvature radius (*R*_*m*_) corresponds to the inverse of the average curvature of the internal Spline. On the other hand, Figure 7 shows a comparison between the real sample and the computational geometry Case D (scaled to the real sample).

$$R_{m}=\frac{1}{\sum_{i=1}^{n}\frac{\kappa_{i}}{n}}$$ (4)

**TABLE 2:** Experimental-numerical comparison of the tissue initial configuration.

	Internal perimeter [mm]	External perimeter [mm]	Mean thickness [mm]	Mean curvature radius [mm]
Experimental	16.044	21.922	0.763	2.480
Case A	14.038	18.727	0.746	2.234
Case B	14.047	18.758	0.746	2.236
Case C	14.130	18.824	0.746	2.238
Case D	14.018	18.773	0.746	2.228

Where *κ*_*i*_ represents the curvature of the spline at each discrete coordinate that forms part of the spline definition, and *n* the number of coordinates.

**FIGURE 8:**
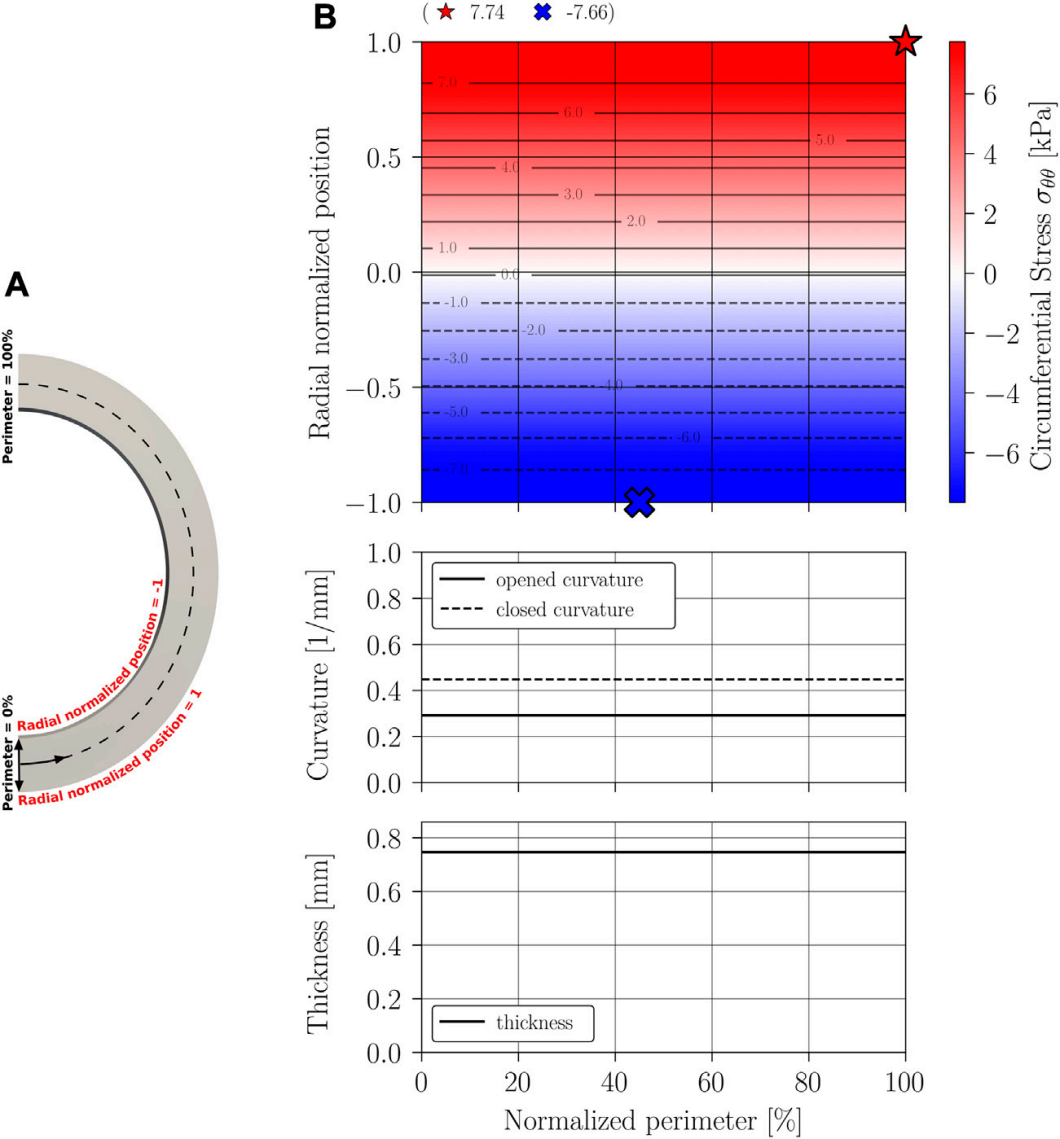
Circumferential stress *σ*_*θθ*_ results on Case A geometry: constant thickness and curvature geometry simplification. **(A)** Closed geometry (symmetry); **(B)** Stress mapping.

**FIGURE 9:**
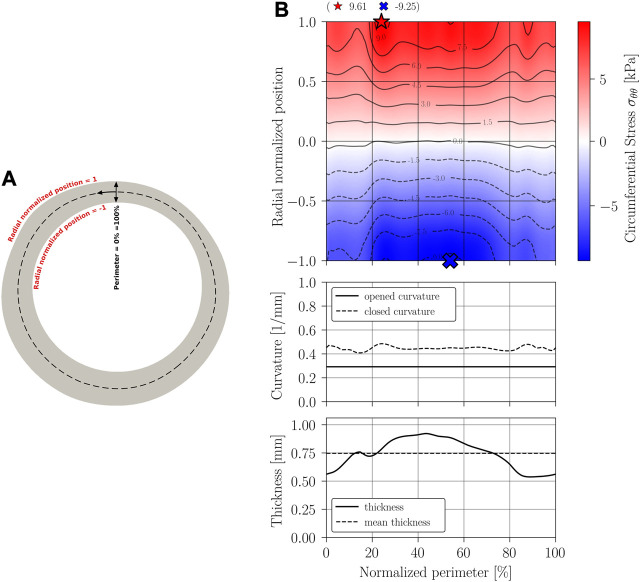
Circumferential stress *σ*_*θθ*_ results on Case B geometry: variable thickness and constant curvature. **(A)** Closed geometry; **(B)** Stress mapping.

**FIGURE 10:**
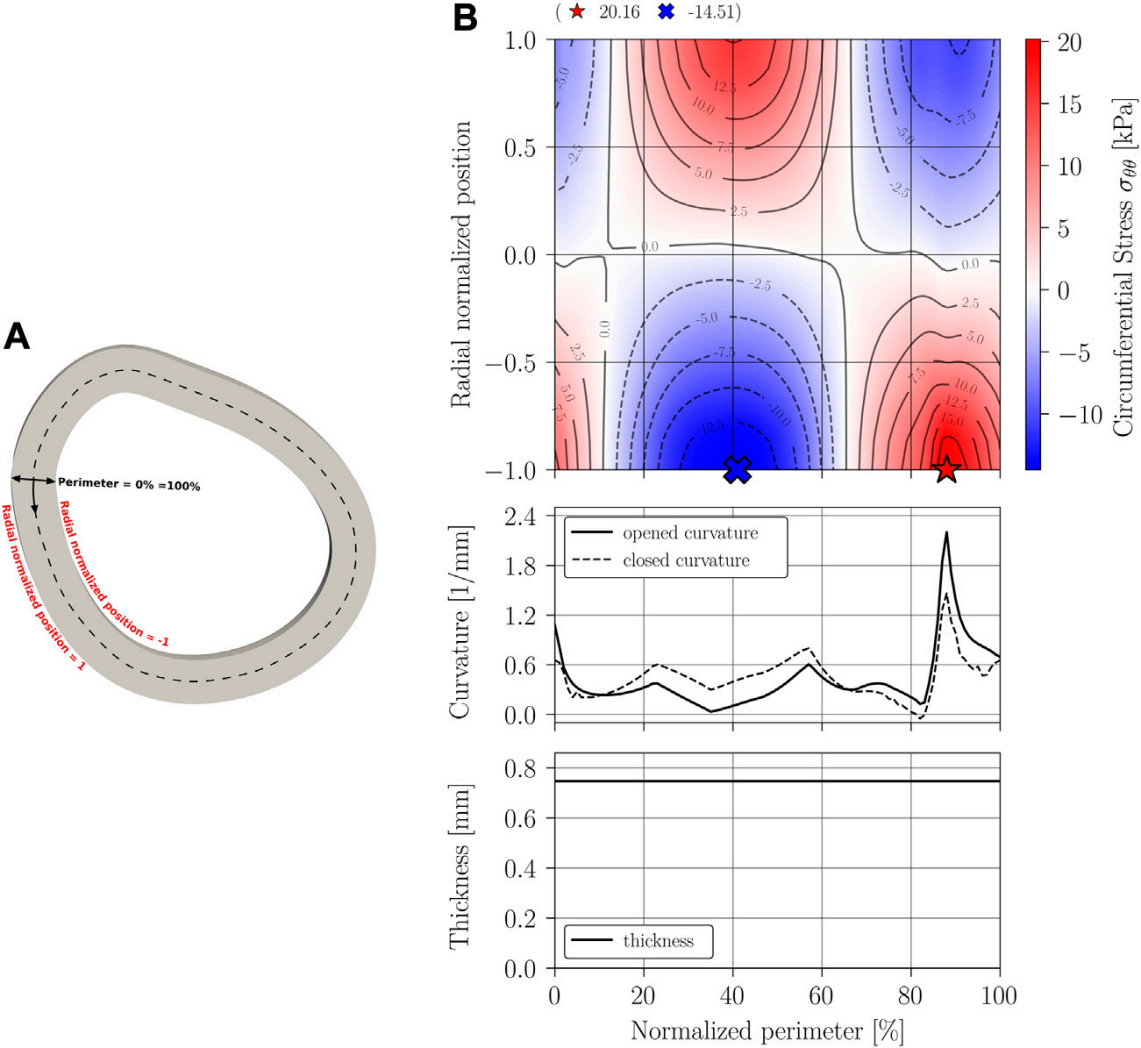
Circumferential stress *σ*_*θθ*_ results on Case C: constant thickness and variable curvature geometry. **(A)** Closed geometry; **(B)** Stress mapping.

**FIGURE 11:**
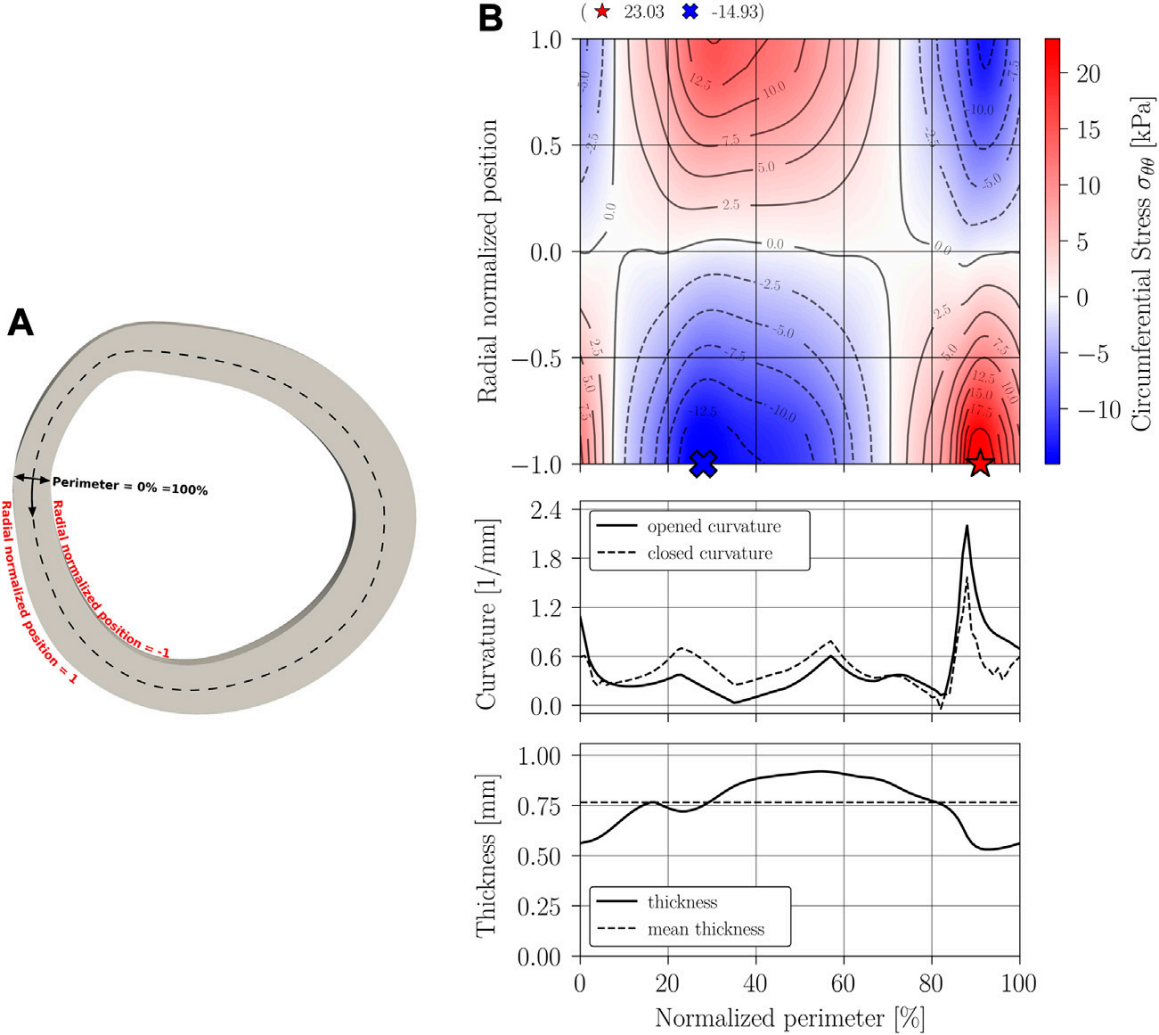
Circumferential stress *σ*_*θθ*_ results on Case D: constant thickness and variable curvature geometry. **(A)** Closed geometry; **(B)** Stress mapping.

The main results of the closing procedure of cases A, B, C, and D are expressed through three graphs: residual circumferential stress, through a contour map; curvature; and thickness. All graphs are presented along the normalized perimeter of the sample as indicated in the schematics Figures 8A, 9A, 10A, 11A.

Figures 8, 9 display the results of the residual stress estimation when the artery’s cross-section is modeled with constant curvature and thickness, and when is modeled with constant curvature and variable thickness.

Figures 10, 11 show the results obtained when considering a stress-free geometry with variable curvature and constant thickness, and variable curvature and thickness, respectively. Both geometries provide a better approximation of the opening phenomenon, with the latter being the most realistic approximation.

#### 3.2.2 Pres-stretch and pressurization

Once the closing procedure is completed and the residual stress field is obtained, the internal pressurization simulation is carried out on each of the three models at pressures of 60, 120, and 150 mmHg, meaning a total of nine FE analyses.

**FIGURE 12:**
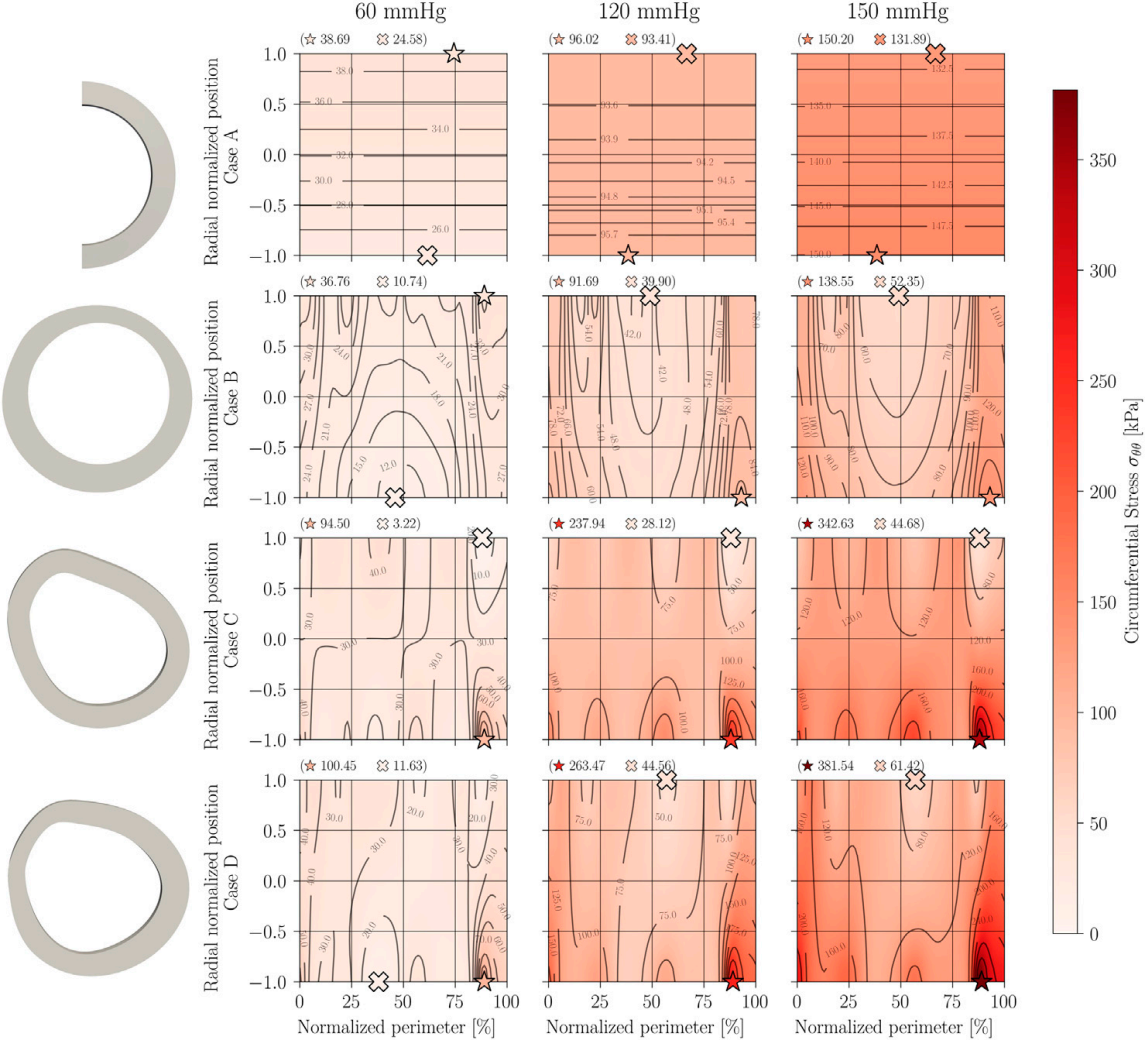
Circumferential stress *σ*_*θθ*_ on pressurization.

Figure 12 displays a matrix-shaped panel with all the results of circumferential stress under pressure loading. The columns are ordered by pressure (ascending towards the right hand), while the rows represent the three geometric models: constant curvature and thickness (case A); constant curvature and variable thickness (case B); variable curvature and constant thickness (case C); and variable curvature and thickness (case D).

## 4 Discussion

In past works, approximations of non-idealized residual stress fields have been made through mathematical formulations, such as the work presented by Sigaeva et al. (2019), which approximates the curvature variability by discretization into sectors of constant curvature. However, the discretization of geometry facilitates the loss of information because the analytical model is not expected to be valid in the vicinity of the curvature transition regions. In this context, an advantage of a numerical formulation lies in obtaining more continuous results, both in terms of stress and strain. Given the high variation in curvature of the selected sample, a numerical formulation can be considered ideal for representing the physical phenomenon. On the other hand, stress results presented through contour maps provide a better understanding of the spatial distribution of residual stresses, as well as facilitating the identification of the most critical areas, unlike studies that have obtained residual stresses in more realistic geometries (Urevc et al., 2016; Heming et al., 2021).

Regarding the results obtained from the geometric reconstruction of the sample, Table 2 suggests that the models perform well from a geometric point of view, with a low difference in geometric aspects between the three models considered and the original sample. It is important to highlight that the average thickness remains practically unchanged in all three cases.

The results of Figure 8B indicate that, when considering an idealized circular geometry, the circumferential stress evolves from a state of compression (inner perimeter) to traction (outer perimeter) in the radial direction, remaining constant along the circumferential direction, that is, an axisymmetric pattern is exhibited. Near the section of the mean radius (normalized radial position of value 0), there is a zone of zero circumferential stress, displaced towards the inner perimeter. The same phenomenon of stress distribution was demonstrated experimentally by Han and Fung (1996), and numerically reported in other studies of this kind such as the one made by García-Herrera et al. (2016).

The curvature of the closed sample remains constant and presents an increase compared to its initial configuration, as it is expected from the classic ring-opening test. Consequently, an increase in curvature with respect to a stress-free configuration is associated with a state of compression in the inner wall.

Results from Figure 9B show that including a real thickness and remaining a constant curvature, the residual stress can be larger than a constant thickness model can predict. The magnitude of residual increases a 24% in tension and a 20% in compression. The residual stress distribution follows the tendency of the Case A model, that means, tension at outer perimeter, compression at inner perimeter, and a zone of transition with zero stress near zero value of radial normalized position (middle perimeter). Although the curvature is initially constant along the perimeter, it can be noted that the curvature is variable at the final configuration, so that, the residual stress does not show a perfect axisymmetric pattern. Despite this, curvature try to reach to the final curvature of the Case A model.

Figure 10B indicates that, when considering the curvature variability in modeling stress-free geometry, a heterogeneous distribution of residual stress occurs, with transitions between compression and traction in both the radial and circumferential directions. This implies the existence of more circumferential stress-free regions within the arterial wall. The magnitudes of the residual stress in this model are widely contrasting compared to the constant curvature model, with an increase of 160% in tension and 89% in compression regarding the stresses of the classical approach. Unlike the residual stress prediction in the constant curvature model, residual stress in a state of tension can be found on the inner perimeter of this model. This could imply that under an internal pressure load, stress concentration in those regions will be strengthened inside the vessel, and therefore, they will have a significant relevance. It should be noted that a localized increase in curvature with respect to the initial configuration produces a state of compression on the inner perimeter, while a decrease implies a state of tension on the inner wall. This third scenario is what leads to inner tensile circumferential stresses in this model, which are observed in the regions ranging approximately from 0% to 10% and 70%–100% in the normalized perimeter. Naturally, the opposite behavior is observed in the outer wall, where the stress field exhibits compressive solicitations in those regions.

The results of variable curvature and thickness geometry (Figure 11B) show a stress distribution locally similar to that of variable curvature and constant thickness geometry. The difference is that the inclusion of thickness modifies the magnitude of the maximum compressive and tensile stresses, resulting in a 198% increase in tension and a 95% increase in compression to the idealized model (case A); a 131% inrease in tension and a 61% increase in tension compared to the case B geometry; and a 14% increase in tension and a 3% increase in compression to the variable curvature and constant thickness model (case C). The large diffece between Case B and D, and the low difference between Case C and Case D models suggests that the incorporation of thickness variability may have less impact than the incorporation of curvature variability. As in the Case B analysis, localized compression is evident within the sample as curvature increases, and tension occurs in the opposite case. The fact that this phenomenon is repeated in all three models implies that the physical phenomenon is dominated by a bending situation (Chuong and Fung, 1986; Urevc et al., 2016; Sigaeva et al., 2019).

These results could indicate that the opening angle by itself is not enough to entirely characterize the physical phenomenon, and moreover, visible changes in the curvature along ring samples may have a strong impact when this parameter aims to establish correlation between conditions or diseases with residual stresses, as is usually utilized in many studies.

From the pressurization simulations presented in Figure 12, it can be established that, despite their low magnitude, residual stresses have a noticeable impact on the distribution of stresses under a pressure load. When considering an idealized initial geometry (Case A) of the chosen sample, the incorporation of residual stresses homogenizes the field of circumferential stresses by reducing the tensile stress induced on the inner wall due to the pressure load. This is because the prior residual compression on the inner wall counteracts part of the tensile stress due to pressure, reducing the difference between the maximum and minimum stress levels within the arterial wall. This homogenization of the stress field has been discussed and demonstrated in several works (Chuong and Fung, 1986; Matsumoto and Hayashi, 1996; Delfino et al., 1997; García-Herrera et al., 2016), where comparisons of pressurization of idealized ring models with and without residual stress have been done analytically and numerically.

When incorporating geometric changes from a realistic geometry, localized stress concentrations can be observed mainly on the inner perimeter. This occurs because of the tensile state provided by residual stress after reconstruction, which increases the effect of the tensile stress due to the application of pressurization on the inner wall. In comparison to an idealized geometry, modeling this specimen with a realistic geometry introduces significant differences between the minimum and maximum stresses found within the arterial wall, and this difference is increased as the value of internal pressure increases.

Although all of these scenarios indicate the importance of evaluating more realistic conditions when dealing with highly non-homogeneous geometries in arterial samples, a remaining analysis related to local material properties should be addressed. This new consideration would be valuable in order to determine if there are regions with altered or damaged elastic properties, that could modify the stress fields from the results presented in this work, which consider constant material properties for the entire sample.

As a final remark, it is important to highlight that there were experimental limitations that should be addressed to get even more realistics results. For example, the sample precondition before mechanical tests as recommended in literature (Carew et al., 2000), and carry out tests with more physiological stress modes, such as biaxial or pressurization tests. This should affect the mechanical characterization, and therefore, the stress-strain prediction of the model.

## 5 Conclusion

Numerical simulations of the ring opening test were used to obtain the residual stresses developed in a lamb abdominal aorta sample that presented a highly non-constant curvature, which is visible to the naked eye. To this end, a geometry digitalization procedure based in splines curves was developed and applied, in order to perform FE analysis that allows to approximate the internal stresses developed in this tissue at physiological conditions.

Four cases were presented and analyzed, focused on geometry parameters such as curvature and thickness. This study shows that geometry highly impacts on residual stress distribution along the thickness and the perimeter of the sample. Thus, the incorporation of curvature and thickness variability are necessary to get more accurate results.

The limitations of this work that should be addressed in further research are mainly related to constitutive modelling, such as, consideration of layer-based laws, viscoelastic effects, active response, and a characterization with tests that represents a more physiological stress state, such as, biaxial or pressurization test. All of these factors could affect the stress distribution field.

## Data Availability

The raw data supporting the conclusion of this article will be made available by the authors, without undue reservation.
